# Role of p27^Kip1^ as a transcriptional regulator

**DOI:** 10.18632/oncotarget.25447

**Published:** 2018-05-25

**Authors:** Oriol Bachs, Edurne Gallastegui, Serena Orlando, Anna Bigas, José Manuel Morante-Redolat, Joan Serratosa, Isabel Fariñas, Rosa Aligué, Maria Jesús Pujol

**Affiliations:** ^1^ Department of Biomedical Sciences, Faculty of Medicine, University of Barcelona, IDIBAPS, CIBERONC, Barcelona, Spain; ^2^ Program in Cancer Research, Institut Hospital Del Mar d'Investigacions Mèdiques (IMIM), CIBERONC, Barcelona, Spain; ^3^ Departamento de Biología Celular, Biología Funcional y Antropología Física and ERI de Biotecnología y Biomedicina, CIBERNED, Universidad de Valencia, Valencia, Spain; ^4^ Department of Cerebral Ischemia and Neurodegeneration, Institut d'Investigacions Biomèdiques de Barcelona, Consejo Superior de Investigaciones Científicas (CSIC), IDIBAPS, Barcelona, Spain

**Keywords:** p27^Kip1^, transcriptional regulation, cancer, neurodegeneration

## Abstract

The protein p27^Kip1^ is a member of the Cip/Kip family of cyclin-dependent kinase (Cdk) inhibitors. It interacts with both the catalytic and the regulatory subunit (cyclin) and introduces a region into the catalytic cleave of the Cdk inducing its inactivation. Its inhibitory capacity can be modulated by specific tyrosine phosphorylations. p27^Kip1^ also behaves as a transcriptional regulator. It associates with specific chromatin domains through different transcription factors. ChIP on chip, ChIP-seq and expression microarray analysis allowed the identification of the transcriptional programs regulated by p27^Kip1^. Thus, important cellular functions as cell division cycle, respiration, RNA processing, translation and cell adhesion, are under p27^Kip1^ regulation. Moreover, genes involved in pathologies as cancer and neurodegeneration are also regulated by p27^Kip1^, suggesting its implication in these pathologies.

The carboxyl moiety of p27^Kip1^ can associate with different proteins, including transcriptional regulators. In contrast, its NH2-terminal region specifically interacts with cyclin-Cdk complexes. The general mechanistic model of how p27^Kip1^ regulates transcription is that it associates by its COOH region to the transcriptional regulators on the chromatin and by the NH2-domain to cyclin-Cdk complexes. After Cdk activation it would phosphorylate the specific targets on the chromatin leading to gene expression. This model has been demonstrated to apply in the transcriptional regulation of p130/E2F4 repressed genes involved in cell cycle progression.

We summarize in this review our current knowledge on the role of p27^Kip1^ in the regulation of transcription, on the transcriptional programs under its regulation and on its relevance in pathologies as cancer and neurodegeneration.

## INTRODUCTION

**Figure 1:**
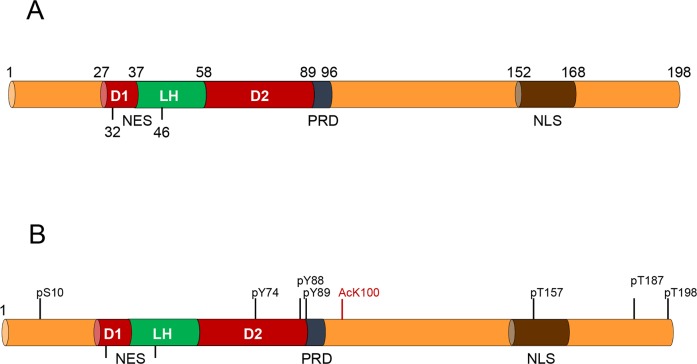
Representation of the key functional domains of p27 along with the most relevant post-translational modifications **(A)** The N-terminal domain of p27 includes the kinase inhibitory domain (KID) that consists of three subdomains: the cyclin-binding subdomain (D1), the Cdk-binding subdomain (D2) and a linker subdomain that joins D1 and D2 (LH). This region also includes a nuclear export signal (NES). In the region subsequent to the KID domain there is a proline rich domain (PRD) (aa 89-96). The C-terminal domain of p27 is an intrinsically disordered region that contains a nuclear localization signal (NLS). **(B)** The most relevant sites of phosphorylation are represented in black. The specific site of acetylation (K100) is marked in red.

**Figure 2:**
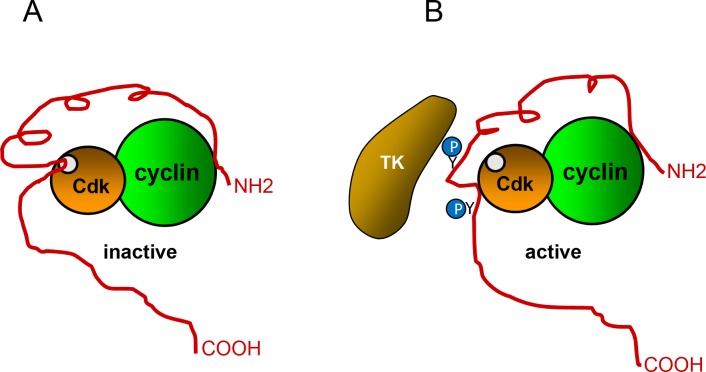
Regulation of p27 activity by phosphorylation **(A)** To inhibit cyclin-Cdk activity, p27 associates with both, the cyclin subunit and the Cdk. A p27 portion is introduced into the catalytic center of the Cdk, This portion competes with ATP thus, blocking the transfer of phosphate to the substrates. **(B)** Phosphorylation of specific tyrosine (Y74 and Y88) residues of p27 by different tyrosine kinases (TK) generates a conformational change that separates the inhibitory region from the catalytic cleavage of the Cdk, leading to its partial activation.

The protein p27^Kip1^ (p27) is a member of the Cip/Kip family of cyclin dependent kinase (Cdk) inhibitors that also includes p21^Cip1^ (p21) and p57^Kip2^ (p57) [1]. The gene encoding human p27 (CDKN1B) is mapped to chromosome 12p13, contains 3 exons and encodes for a protein of 198 aa [2, 3]. These three proteins lack stable tertiary structure in isolation and are classified as intrinsically disordered proteins [4, 5]. Despite of that, they have several structured regions in their N-terminal domains [6]. These regions are included in the kinase inhibitory domain (KID) that is conserved between these proteins. The KID consists of three subdomains: the cyclin-binding subdomain (D1), the Cdk-binding subdomain (D2) and a linker subdomain that joins D1 and D2 (LH) [7, 8]. In p27 the KID domain includes aa 28-89 and contains a nuclear export signal (NES) (aa 32-46) (Figure 1A). In order to inhibit Cdk, a portion of the D2 subdomain is introduced into the catalytic center of the Cdk. This portion competes with ATP thus blocking the transfer of phosphate to the substrates (Figure 2A) [9]. Whereas D1 and D2 are mostly unfolded in isolated p27 they fold after binding to cyclins and Cdks. In contrast, LH is already structured as an α-helix before association with cyclin-Cdk and remains similarly folded after binding. In the region subsequent to the KID domain there is a proline rich domain (aa 90-96) known to interact with a variety of proteins as for instance the adaptor protein Grb2 [10]. The C-terminal domain (CTD) of p27 is an intrinsically disordered region that associates with a growing number of different proteins and contains a nuclear localization signal (NLS) (aa 152-168) (Figure 1A).

The cellular levels of p27 are regulated by both transcriptional and post-transcriptional processes. A number of transcription factors (TFs) including FKHR-L1, AFX, FOXO, Sp1, E2F1, and Menin have been identified as transcriptional activators of p27, whereas, in contrast, c-myc, Id3 and Ap1 act as transcriptional repressors [11–17]. However, it is assumed that the relevance of transcriptional regulation of p27 is low whereas in contrast, the main role in regulating cellular p27 levels is carried out by the control of its degradation [18, 19]. Specifically, p27 is mostly controlled by ubiquitin dependent-proteolysis which requires its phosphorylation or acetylation. Acetylation of p27 at K100, at mid G1 phase of the cell cycle, by the acetyl transferase p300/CBP associating factor (PCAF), has been shown to induce its ubiquitylation and subsequent degradation via proteasome in the cell nucleus [20] (Figure 1B). The phosphorylation of T187 by Cyclin-Cdk2 complexes also induces its degradation in the nucleus. Specifically, this phosphorylation is recognized by the F-box protein Skp2, a subunit of the SCF E3 ubiquitin ligase that triggers its ubiquitylation and subsequent degradation via proteasome (Figure 1B) [21]. Interestingly, phosphorylation of specific tyrosine residues in the D2 subdomain (Y74 and Y88 in p27), by different tyrosine kinases, including the Src family, allows the partial activation of the Cdk in the trimeric complexes formed by cyclin, Cdk and p27 due to a conformational change of this p27 domain (Figure 2B) [22–26]. The partial activation of the Cdk allows to this Y-phosphorylated p27 to be efficiently phosphorylated at threonine 187 by the activated Cdk, which in turn promotes its Skp2 dependent degradation. Thus, specific tyrosine phosphorylation of p27 stablish a direct link between p27 Cdk-inhibitory activity and stability [22]. Interestingly, something equivalent happens with p21 [27].

Phosphorylation of p27 at S10 induces its translocation from nucleus to the cytoplasm (Figure 1B). This phosphorylation, mediated by different kinases as Cdk5, Akt or Kis, facilitates its binding to the carrier protein CRM1 that transports p27 to the cytoplasm [28]. Once in the cytoplasm S10-phosphorylated p27 can be retained there by its phosphorylation at T157 and T198 by different kinases like Akt, SGK or AMPK [11]. These phosphorylations are recognized by 14-3-3 proteins that sequesters p27 in the cytosol [29]. Interestingly, T157 is located in the NLS of p27 and its phosphorylation may impair its transport to the nucleus (Figure 1B). Once in the cytoplasm phosphorylated-p27 can be stabilized or in contrast, it can be degraded by the E3 ubiquitin ligases KPC1 and KPC2 [11].

Recent evidence indicate that p27 is involved in the regulation of cytoskeleton reorganization and gene expression [30]. It has also been reported that p27 deregulation (decreased levels or cytoplasmic localization) might be involved in relevant pathologies as cancer and neurodegeneration [3, 31, 32]. In this review we aim to briefly summarize the role of p27 as a transcriptional regulator and its implication in these pathologies.

## P27 REGULATES THE ACTIVITY OF MEMBERS OF THE CDK FAMILY

All the members of the Cip/Kip family have the ability to associate with a number of different cyclin-Cdk complexes and act by binding to both cyclins and Cdks [9]. The classical role of the Cip/Kip members is to inhibit the activity of most cyclin-Cdk complexes involved in cell cycle progression [33].

Cyclin-dependent kinases (Cdks) are serine/threonine kinases whose activity depends on their association with specific regulatory subunits named cyclins [34]. Cdks include 20 kinases named as Cdk1 through Cdk20 [35]. These kinases were initially described as essential drivers of cell cycle progression [36, 37]. However, research performed during the last decades provided evidence that Cdks are also involved in gene transcription, DNA damage repair, cell differentiation and metabolism. Despite of these diversity of functions, Cdks have been classically classified into two main groups corresponding to Cdks involved in cell cycle regulation (Cdk1, Cdk4 and Cdk5 subfamilies) and to Cdks involved in transcription (Cdk7, Cdk8, Cdk9, Cdk11 and Cdk20 subfamilies) although these activities are frequently combined in many family members [38]. Cdks are primarily activated by their binding to regulatory subunits named cyclins because their protein levels oscillate during the cell cycle [39, 40]. The cyclin family contains approximately 29 members in humans with a high spectra of molecular weights (35-90 kDa). Most of the Cdks bind one or a few cyclins consistent with specialized functions acquired during evolution [41].

Cdk activity is regulated by different mechanisms that include: 1) association with cyclins that activate Cdks, 2) an activating phosphorylation, 3) inhibitory phosphorylations, 4) inactivation by acetylation, and 5) association with inhibitory proteins. As mentioned above, the association of a Cdk with its cyclin partner is needed for a minimal activity. This activity is enhanced by the specific phosphorylation of a threonine residue (T160 in Cdk2) by the cyclin H-Cdk7 complex (Cdk-activating kinase (CAK)) that stabilizes the activated form of the Cdk [42]. Moreover, Cdk activity can be inhibited by phosphorylation of specific residues (Thr14 and Tyr 15 in Cdk2) by Wee1 and Myt1 kinases [43]. These phosphates can be eliminated by the action of phosphatases of the Cdc25 family thus re-inducing Cdk activity [44]. It has also been reported that the acetyltransferases GCN5 and PCAF, regulate CDK9 function by specifically acetylating the catalytic core of the enzyme and, in particular, a lysine that is essential for ATP coordination and the phosphotransfer reaction. This acetylation inhibits Cdk9 activity [45]. Because this lysine is highly conserved in all the Cdks, it has been postulated that this may be a general mechanism involved in Cdk inhibition. In fact, a report showing that PCAF acetylates an equivalent lysine in Cdk2 inhibiting its activity supports this possibility [46]. Cdk activity can also be regulated by the association to inhibitory proteins that belongs to two families, the Ink4 family and the Cip/Kip family. The ink4 family includes p16^Ink4a^, p15^Ink4b^, p18^Ink4c^ and p19^Ink4d^ [47]. These members act by specifically associating with Cdk4 and Cdk6 and thus blocking their interaction with the D-type cyclins. The aforementioned Cip/Kip family includes p21, p27 and p57.

The extracellular signals regulating cell cycle trigger the activation of different pathways that finally act by regulating cyclin-Cdk activity through any of these different mechanisms. Also the intracellular cell cycle checkpoints act controlling the activity of Cdks through these mechanisms.

## TRANSCRIPTIONAL REGULATION MEDIATED BY P27

### Transcriptional regulators as substrates of Cdks

In the current model of cell cycle regulation, the main targets of the G1 cyclin-Cdk complexes (Cyclin D-Cdk4/6 and cyclin-Cdk2) are the members of the pocket proteins family: the retinoblastoma protein (pRB), p130 and p107 [48]. During the G1 phase of the cell division cycle, important genes involved in DNA replication (S phase) and in the subsequent phases of the cell cycle are repressed by complexes including pocket proteins and different members of the E2F transcription factors (TFs) family associated to DPs [49]. Specifically, the complexes pRB-E2F (1-3) and p130-E2F4 are associated to E2F binding sites on the chromatin regulatory regions of specific genes in quiescent cells. After proliferative activation active Cdk4/6 mono-phosphorylate both pRB and p130 [50, 51]. Later on, cyclin-Cdk2 complexes multi-phosphorylate pRB and p130 leading to their separation from the repressive complexes [50, 51]. Then, the repressor E2F4 moves out of the chromatin and translocate to the cytoplasm [52] whereas in contrast, the activators E2F1-3 remain in the chromatin and promote gene expression [53, 54].

In addition to these main targets, cyclin-Cdk complexes are able to phosphorylate a number of TFs thereby linking cell cycle and transcription [55]. Among them, it is specifically interesting the phosphorylation of FOXM1 and B-Myb since these TFs associate with the MuvB repressor complexes that regulate the expression of G2/M genes during cell cycle [49, 55]. TFs as Smad3, ID2, UBF, NFY and Myc are also phosphorylated by cyclin-Cdk complexes regulating important cellular functions [56–60]. Since p27 regulates the activity of cyclin-Cdk complexes that phosphorylate these TFs, it becomes an important player in regulating transcription.

There are several Cdk subfamilies that are directly involved in the regulation of transcription (Cdk7, Cdk8, Cdk9, Cdk11 and Cdk20 subfamilies). The main role of these Cdks is to regulate the activity of RNA polymerase II (RNAPII) and this is performed by phosphorylating specific sites of the C-terminal domain (CTD) of the largest subunit (Rpb1) of RNAPII. Also cell cycle-related kinases Cdk1 and Cdk2 phosphorylate the CTD [61, 62]. Despite the ability of p27 to regulate the activity of these transcriptional Cdk subfamilies still remains obscure, its ability to inhibit Cdk1 and Cdk2 implies the direct regulation of transcription through this mechanism.

### p27 as a transcriptional regulator

Quiescent cells have high levels of p27 in the nucleus. However, when cells are induced to proliferate, most of this nuclear p27 translocate into the cytoplasm where it is subsequently degraded. These facts suggested that p27 was playing a role in the nucleus of quiescent cells that had to be related to the inhibition of cell division cycle since proliferating cells needed to reduce or eliminate this nuclear p27. The identification of several chromatin associated proteins, as for instance the transcriptional co-activator PCAF, as p27-binding proteins strongly supported the putative role of p27 in the regulation of transcription [20]. Previous evidences already suggested the participation of p27 in transcription. It was shown that the association of p27 with the TF neurogenin-2 induces its stabilization and the differentiation of neural progenitors in the cortex [63]. It was also reported that p27 associates with the promoter of the Twist1 gene repressing its transcription [64]. The implication of p21, another member of the Cip/Kip family of Cdk inhibitors, in the regulation of transcription also reinforced the putative role of p27 as a transcriptional regulator. It was demonstrated that p21 interacts with and regulates the activity of various TFs as NF-κB, Myc, E2F, STAT3 and estrogen receptor [65–67]. Expression microarray analysis confirmed that over-expression of p21 induced the repression of genes involved in cell cycle progression [68–70]. Interestingly, p21 associates with and regulates the activity of transcriptional co-activators, with acetyltransferase activity, as p300/CBP [71] thus, mediating transcriptional regulation also through this interaction [67]. A general correlation between the cell cycle dependent element (CDE) and the cell cycle gene homology region (CHR) sequences with the p21 inhibitory effects was also reported thus leading to the proposal that transcriptional regulation by p21 can be mediated by these chromatin sequences [70, 72].

### p27 associates with chromatin and regulates transcription

The nuclear localization of p27 in quiescent cells and its interaction with chromatin-bound proteins suggested that p27 could be associated with chromatin and there to play a role as a transcriptional regulator. ChIP on chip analysis performed in quiescent NIH3T3 cells and directed to identify p27-binding sites (p27-BSs) on gene promoters firstly demonstrated that p27 specifically associates with 427 gene promoters. This association was further validated in a number of genes by ChIP followed by qPCR [73]. Gene Ontology (GO) classification of these putative p27-target genes (p27-TGs) revealed that p27 could participate in the regulation of genes involved in important cellular functions as RNA processing and splicing, translation, cell division cycle, respiration and Golgi vesicle transport. The evidence showing that p27 could regulate the expression of these genes were obtained from expression microarray analyses performed in quiescent mouse embryonic fibroblasts (MEFs) from WT and p27 knock out (p27KO) animals. Results revealed that a significant number of the previously identified p27-TGs were up-regulated in p27KO cells indicating a transcriptional repressor role of p27 [73]. MEFs harboring different p27 mutants unable to associate with cyclin–Cdk complexes (the p27^Δ51^ mutant that has a deletion of the first 51 aa and the p27^CK−^ mutant including mutations in the regions of interaction with cyclins and Cdks [74, 75]) also showed a significant up-regulation of p27-TGs [73]. Interestingly, most of the up-regulated genes overlapped in these different cell types [73]. In all these cells, Cdk activities are high since p27 is not operative as a Cdk inhibitor. Thus, these results indicate that the Cdk inhibitory ability of p27 is essential for the repression of transcription. Intriguingly, cells with the p27^CK−^ mutant additionally showed a significant number of down-regulated p27-TGs that were not decreased in p27KO or p27^Δ51^ cells [73]. These results revealed that the transcriptional regulatory role of p27 can proceed through different mechanisms but that inhibition of cyclin-Cdks is essential for gene repression in at least a significant number of p27-TGs [73].

Two different articles have reported ChIP-seq analyses that have been performed to identify the p27-BSs in the chromatin of two different cellular types. One has been performed in quiescent HCT116 colorectal cancer cells [76] and the other one in quiescent MEFs [77]. In HCT116 cells, 1981 specific p27-BSs that were annotated to 1012 genes were identified (Table 1). Similarly, in MEFs, 1839 p27-BSs were detected. In this case they were annotated to 1417 genes. These results indicate that in some cases more than one p27-BSs was annotated to a specific gen. These studies also revealed that in both cellular types only a small number of p27-BSs were on gene promoters while most of them were in distal intergenic (74% and 64%) or intronic regions (24% and 31%). Another interesting observation was that a significant number of p27-BSs (~40% in both cases) were close to pseudogenes or sequences of non-coding RNAs suggesting a role of p27, not only in the transcriptional regulation of protein encoding genes, but also of pseudogenes and non-coding RNAs [76, 77]. Specifically, a significant number of microRNAs (Mirs) were annotated to p27-BSs in both cellular types. Most of these Mirs are different in these cellular types being mir-214 the only one shared between the two cell types. Mir-214 is involved in tumorigenesis in different types of cancer and it has been proposed to be useful as a cancer biomarker [78]. Most of the other Mirs that can be putatively regulated by p27 (unpublished results) are also involved in cancer. Interestingly, also a number of small nucleolar RNAs (snoRNAs) were annotated to p27-BSs in both types of cells. They belong to the two groups (H/ACA and C/D) that promote pseudouridylation and methylation, respectively of the pre-ribosomal RNA but also of pre-mRNA [79]. It has been reported that deregulation of different snoRNAs expression is involved in neurodegeneration and cancer [80, 81].

**Table 1:** ChIP-seq data obtained from experiments in two different cell lines using anti-p27 antibodies

Cell type	p27-BSs	Genes	Distal intergenic	Intronic	promoters
HCT116	1981	1012	74%	24%	0.3%
MEFs	1839	1417	64%	31%	0.8%

Table summarizes the cell types used, the number of p27-binding sites (p27-BSs) identified, the number of genes to which these p27-BSs were annotated and the percentage of p27-BSs associated to distal intergenic, intronic or promoter regions.

**Table:** 

Cell type	Protein	ncRNAs	Pseudogenes
HCT116	59%	23%	18%
MEFs	60%	28%	12%

Table summarizes the cell types used in the ChIP-seq analysis and the biotypes corresponding to the annotated genes. Specifically, the table includes the percentages of annotated protein encoding genes (protein), non-coding RNAs (ncRNAs) and pseudogenes.

These ChIP-seq analyses also revealed that 7SK RNA can be putatively regulated by p27. This RNA regulates the activity of P-TEFb that stimulates transcriptional elongation [82]. Finally, the five major spliceosomal RNAs (U1, U2, U4, U5, U6 and U7) [83] are also found annotated to specific p27-BSs, suggesting that p27 might regulate splicing by controlling the expression of these RNAs. Thus, the putative regulation of these different types of non-coding RNAs by p27 has to be taken into account in the next future to better understand the role of p27 in tumorigenesis, neurodegeneration and perhaps other pathologies.

**Figure 3:**
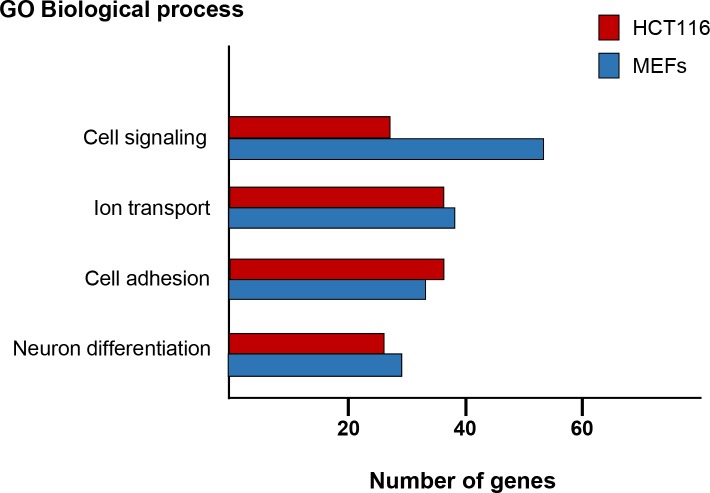
Gene ontology analysis of protein encoding genes with p27-BSs in their proximity ChIP-seq analysis using p27-antibodies were performed in HCT116 and MEFs cells. The Database for Annotation, Visualization and Integrated Discovery (DAVID) program was used to define biological processes enriched in the protein encoding genes putatively regulated by p27. The number of genes included in each biological process is represented.

Remarkably, GO classification of the protein encoding genes putatively regulated by p27 revealed that in both cellular types, the four more significant biological processes were cell adhesion, neuron differentiation, cell signaling and ion transport (Figure 3). The transcriptional regulation of a number of these putative p27-TGs by p27 was further validated by qPCR in both cellular types.

Taken together all these results indicate that the role of p27 in transcription is mostly performed by its association to chromatin regions distal from gene promoters and intronic regions. The genes putatively regulated by p27 through the interaction with these regions are preferably involved in the four biological processes mentioned above. However, despite the global association of p27 with gene promoters seems to be limited, the ChIP on chip analysis performed in NIH3T3 cells indicates that several relevant cellular functions as cell cycle progression, mRNA processing and splicing, translation and respiration are regulated by the p27 association with specific gene promoter sequences. A possible interpretation of these data may be that different sets of genes can be regulated by the association of p27 to different chromatin domains.

### Specific TFs mediate the transcriptional regulatory role of p27

**Figure 4:**
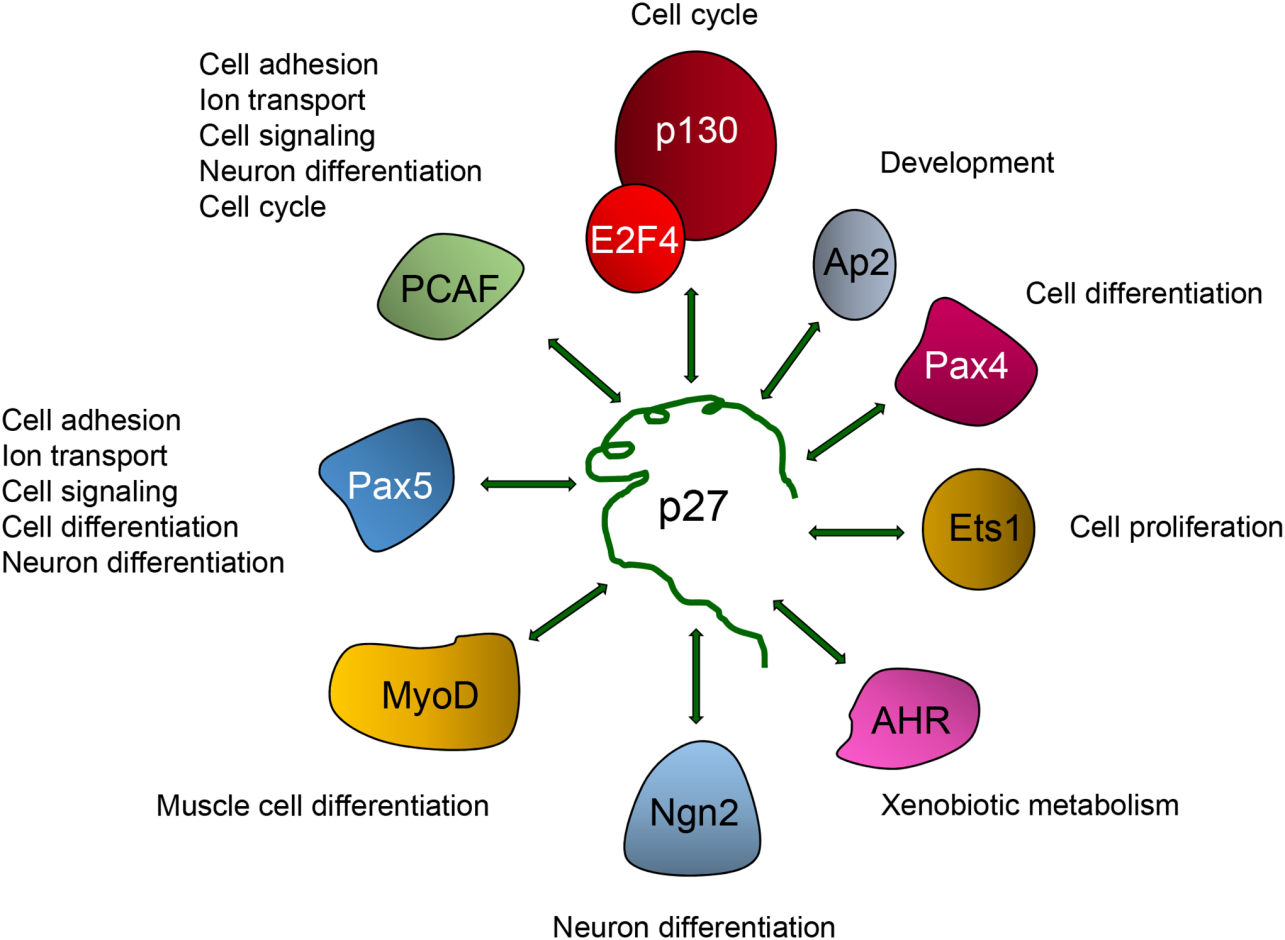
Regulation of transcriptional activity by p27 is mediated by its association with different transcriptional regulators To regulate transcription p27 associates with different transcriptional regulators including TFs as p130/E2F4 complexes, Ap2, Pax4, Ets1, AHR, Neurogenein-2 (Ngn2), MyoD and Pax5. It also interacts with the transcriptional co-activator and acetyltransferase p300/CBP associating factor (PCAF). The biological functions putatively regulated by these TFs and p27 are indicated.

There is no evidence for p27 directly associating with DNA, thus its interaction with the chromatin has to be through chromatin-binding proteins. The identification of the p27-binding proteins that mediate its association with chromatin was firstly achieved by sequence analysis of the p27-BSs on the promoters detected by ChIP on chip [73]. This analysis was directed to determine whether in these p27-BSs there were consensus sequences for TFs. Results revealed that these p27-Bs were enriched with regions able to associate with E2F4 and several members of the ETS family of TFs as for instance ETS1 and GABP. Immunoprecipitation (IP) and ChIP experiments further confirmed that actually p27 associate with specific gene promoters through its binding to E2F4 or ETS1 [73]. Subsequent sequence analysis of the p27-BSs identified by ChIP-seq in HCT116 cells showed that Pax5 could also be a partner of p27. The interaction of PAX5 with p27 was further validated by IP [76]. Finally, sequence analysis of the p27-BSs identified by ChIP-seq in MEFs revealed enrichment of several TFs as AHR, Ap2α and Ap2γ, Pax5, Pax4, and MyoD, among others. The association of p27 with some of these TFs was subsequently validated by IP [77]. Thus, p27 plays a role in the regulation of transcription by associating with a significant number of different TFs (Figure 4). Probably, the complexes of p27 with each one of these TFs may be involved in the transcriptional regulation of specific subset of genes. For instance, the association of p27 with E2F4 or with ETS on specific gene promoters can be involved in the transcriptional regulation of G1/S cell cycle related genes [73] whereas its association with Pax5 in distal intergenic regions can participate in the regulation of genes involved in cell adhesion, cell signaling, ion transport and neuron differentiation [76].

Interestingly, the analysis of the p27-TGs, identified in the ChIP on chip studies, whose expression is altered in cells lacking p27 or in cells with the different p27 mutants gave interesting information [73]. It was observed that in p27KO MEFs the p27-TGs that were up-regulated were those mediated by ETS TFs. In contrast, those repressed by p130/E2F4 were not modified in these cells. These data suggest that p27 is an inhibitor of ETS activity and thus, in the absence of p27, ETS can activate transcription. Intriguingly, in contrast to that observed in p27KO cells, in p27^Δ51^ and p27^CK−^ - MEFs the genes repressed by p130/E2F4 were up-regulated [73]. These results indicate that the presence of the carboxyl moiety of p27 in the cells is necessary to activate the expression of p130/E2F4 dependent genes [73]. Another interesting result is that in p27^CK−^ - MEFs a significant number of p27-TGs was down-regulated. Remarkably, these down-regulated genes were ETS-dependent [73]. Surprisingly, this repression only happened in these cellular type, since p27KO and p27^Δ51^ did not show the down-regulation of these p27-TGs [73]. These results can be interpreted as that the first 51 aa contain a co-repressor sequence that when lost facilitates transcription (by eliminating the sequence, in the case of p27^Δ51^ or when p27 is absent in the p27KO). In contrast, when this sequence is present (p27WT) and able to associate the cyclin and Cdk complexes, transcription is temporally regulated [73]. However, in the case of p27^CK−^, it may be postulated that despite the first 51 aa are present, the mutated form of this fragment, that prevents its association with cyclin-Cdks, might adopt a conformation that totally unable the accessibility of cyclin-Cdk complexes to phosphorylate the transcriptional regulators on the promoters. In this case it additionally plays a role as a strong repressor.

### p27 regulates transcription of p130/E2F4 repressed genes

Detailed investigations have been performed to analyze the mechanisms by which p27 regulates transcription through E2F4. In quiescent cells E2F4 mainly associates with p130 to form transcriptional repressor complexes on promoters of genes involved in cell cycle progression [48, 84, 85]. These complexes additionally include several co-repressors as different HDACs and mSin3A [86, 87]. In these complexes E2F4 directly associates with specific DNA sequences although its interaction requires p130. In the absence of p130 (that does not directly bind to DNA) E2F4 cannot interact with these specific DNA sequences [87]. These complexes mainly act by repressing, during the G1 phase of the cell cycle, the expression of genes encoding proteins needed for DNA replication and the subsequent phases of the cell cycle. However, in cells suffering DNA damage, these complexes might also act by repressing G2/M genes [88, 89] and genes involved in DNA repair as RAD51 and BRC1 [88, 90]. Moreover, other genes not directly involved in cell cycle progression as for instance Sox2 and α-synuclein (α-SYN) can be also repressed by these complexes [91, 92].

IP experiments demonstrated that p27 interacts with members of these complexes as p130, E2F4, several HDACs and mSin3A [73]. Moreover, it has also been shown that p27 directly interacts with E2F4 and p130 through specific domains located in its carboxyl moiety [73]. These interactions allow p27 to participate in these repressor complexes on the promoters of specific target genes. As mentioned above, p130 is a key protein to maintain these repressor complexes on the promoters since the interaction of E2F4 with DNA depends on p130. This is the reason why phosphorylation of p130 by specific cyclin-Cdk complexes during G1 promotes the disruption of p130-E2F4 interaction thus allowing E2F4 to move out of the promoters and facilitating transcription.

**Figure 5:**
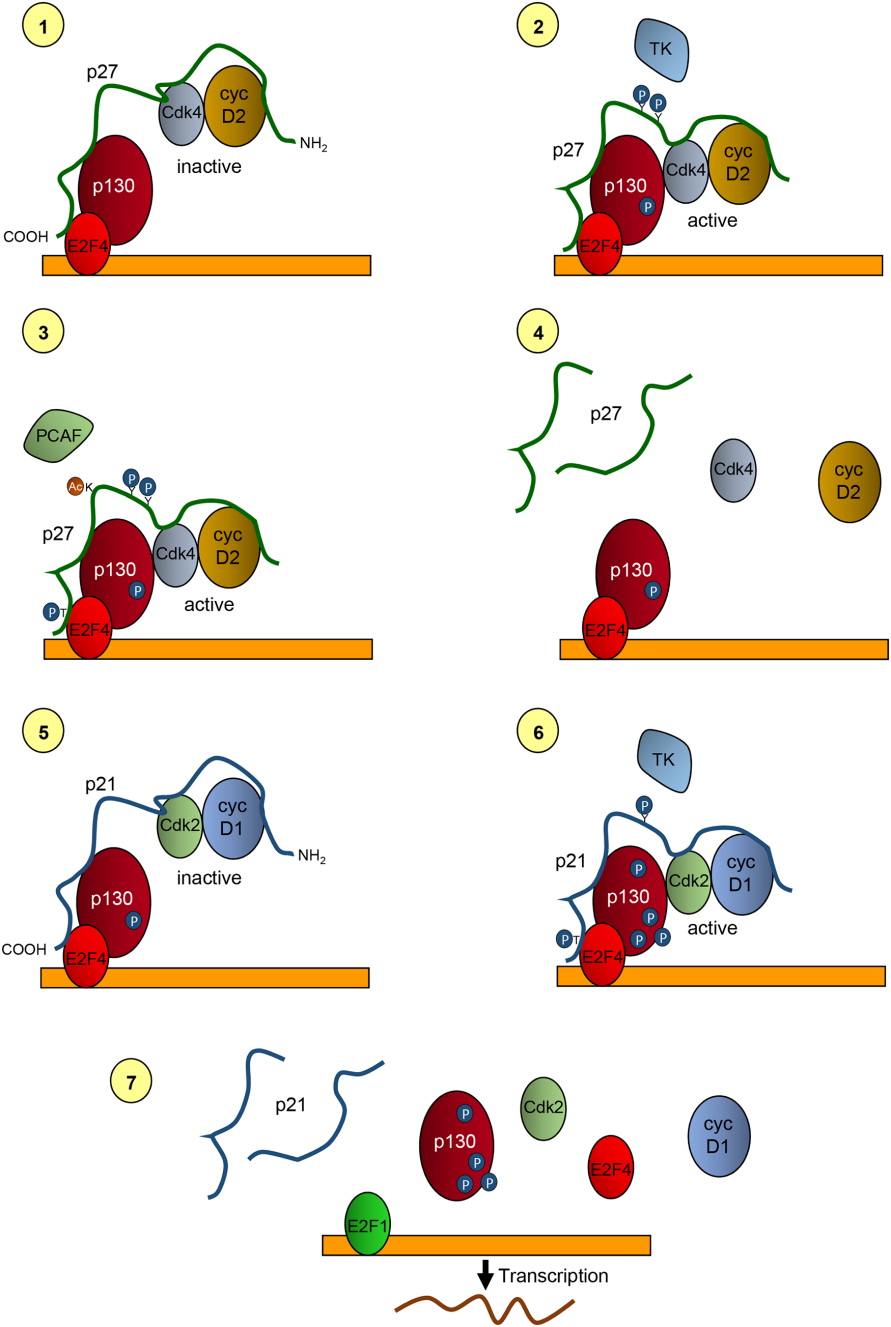
Model of the role of p27 and p21 in the transcriptional regulation of p130/E2F4 repressed genes In quiescent cells p27 is associated with p130 and E2F4 on the promoters of target genes by its COOH- moiety. It is also bound to cyclin D2/3 and Cdk4 by is NH2-half. At this stage, Cdk4 is inactive due to its inhibition by p27 **(1)**. At early-mid G1 phase of the cell cycle p27 is phosphorylated by tyrosine kinases at residues Y74 and Y88. These phosphorylations allow retrieving Cdk activity and thus Cdk4 can phosphorylate p130 **(2)**. Moreover, Cdk4 phosphorylate T187 of p27 facilitating its degradation. Degradation is also stimulated by the acetylation of K100 by the acetyltransferase and transcriptional co-activator PCAF **(3)**. Degradation of p27 leads to the removal of cyclin D2/3 and Cdk4 from the promoters **(4)**. After that, p21 associates with p130/E2F4 by its carboxyl terminus and recruits to the promoters cyclin D1-Cdk2 complexes that are associated with its NH2-moiety **(5)**. After phosphorylation of the Y77 (in human) of p21, Cdk2 becomes activated and able to multi-phosphorylate p130. Cdk2 also phosphorylates S130 of p21 leading to its degradation **(6)**. Phosphorylation of p130 and degradation of p21 disrupt the repressive complexes thus, allowing the action of activator transcription factors as E2F1 that initiate transcription of target genes **(7)**.

Interestingly, the association of p27 with the promoters depends on p130. In fact, in p130 KO cells, p27 is not able to interact with these regulatory regions [73]. This association that is through its carboxyl tail, permits p27 to have its NH2 regions, involved in the interaction with cyclins and Cdks, accessible to associate with these complexes. This is compatible with the simultaneous association of p27 with the promoters through p130/E2F4 by its COOH tail and with cyclin-Cdk by its NH2 terminal domain (Figure 5). Since p130 is a classical substrate of Cdks it has been postulated that in fact, one of the roles of p27 could be to recruit cyclin-Cdk complexes on the promoters of target genes at early-mid G1. This recruitment would allow to place these complexes very close to its substrate p130 and thus facilitating its phosphorylation when the Cdk becomes active (Figure 5). The fine regulation of cyclin-Cdk activity by p27, on depending of its tyrosine phosphorylation status, would also precisely define the timing of Cdk activation and as a consequence the moment of the cell cycle in that p130 should be phosphorylated.

ChIP experiments performed at different times after proliferative activation allowed to generate a model describing the basic mechanisms through which p27 regulates the transcription of genes repressed by p130/E2F4 complexes during the G1 phase of the cell cycle [93]. Since these genes are key for the progression of proliferation this mechanism provides fundamental data to understand the role of p27 in this process. We describe here this model as illustrated in Figure 5.

In quiescent cells, p27 is associated with the promoters of target genes bound directly to E2F4 and p130 by its carboxyl terminus. Simultaneously, cyclin D2/D3-Cdk4 are bound to the KID domain of p27, sited on its amino moiety. At that time, Cdk4 activity is inhibited (Figure 5.1). When cells are induced to proliferate by a mitogenic stimulus, members of the Src protein kinase family or other tyrosine kinases can phosphorylate p27 at Y74 and Y88, also when it is associated with cyclin-Cdk complexes [22]. These phosphorylations induce a conformational change that reduce the inhibitory capacity of p27, thus allowing cyclin D2/D3-Cdk4 complexes to be active, despite being bound to p27 [94–96]. Active Cdk4 associated with p27 located on the promoters of p27-TGs could then phosphorylate p130. This phosphorylation primes p130 for further phosphorylation by Cdk2. (Figure 5.2). Once p130 has been phosphorylated at mid-G1 phase, cyclin D2/D3-Cdk4 complexes must be removed from the promoters to allow the cyclin-Cdk2 entry to subsequently phosphorylate p130.

Removal of cyclin D2/D3-Cdk4 complexes from the promoters can be achieved by degrading p27, since these complexes are associated to the promoters through p27. Several mechanisms can participate in this elimination of p27. It has been shown that phosphorylation in Y74 and Y88 of p27 by tyrosine kinases allows the activation of the associated cyclin D-Cdk4 complexes that furtherly can phosphorylate T187 of p27 inducing its subsequent degradation [22]. Additionally, it has also been shown that, at G1, the acetyl transferase PCAF acetylates p27 at its K100 inducing its ubiquitylation and subsequent degradation (Figure 5.3) [20]. It has been recently reported that PCAF can also facilitate the nuclear translocation of the Cdk4/6 inhibitor p16^Ink4a^ and inducing Cdk4 inhibition [97]. Thus, a combination of these mechanisms induces p27 degradation and Cdk4 inactivation at mid G1. This p27 degradation facilitates the release of cyclin D2/D3-Cdk4 from the promoters, although p130/E2F4 still would remain there since the association of E2F4 with the promoters depends on p130 (Figure 5.4).

The analysis of the mechanism of how cyclin-Cdk2 complexes are incorporated to the promoters revealed that p21 plays an important role in this process [93]. In fact, when p27 moved out from the promoters at mid-late G1, p21 associates with p130/E2F4. Remarkably, the association of cyclin D1 and Cdk2 with the promoters was also observed at that time (Figure 5.5). In fact, the association of cyclin D1-Cdk2 with the promoters is depending on p21 and p21KO cells are not able to incorporate them on these specific regulatory regions [93]. Cyclin D1-Cdk2 is a non-canonical complex that has been reported to exist in many different cellular types [98–101]. Interestingly, it has been shown that the association and activation of cyclin D1-Cdk2 complexes are highly dependent on p21 that acts as an assembly factor. In contrast, p27 is a very poor assembly factor for these complexes [102]. All these results indicate that p27 and p21 collaborate in the regulation of transcription of genes repressed by p130-E2F4 acting as modulators of Cdk activity on the promoters.

At mid-late G1 of the cell cycle p27-cyclin D2/D3-Cdk4 complexes are substituted by p21-Cyclin D1-Cdk2 (Figure 5.5). At that point, Cdk2 is inactivated by p21, but the subsequent phosphorylation of p21 at Y77 (in human cells) by different tyrosine kinases induce, similarly to p27, a reduction of the capability to inhibit Cdk2 (Figure 5.6) [27]. Thus, the activated Cdk2 on the promoters can then multi-phosphorylate p130. Additionally, Cdk2 phosphorylates p21 at S130 that induces its degradation [27]. These phosphorylations induce the disruption of the repressor complexes and as a consequence the promoter is free to bind transcriptional activators as E2F1 that will induce the transcription of target genes involved in DNA replication and cell cycle progression. (Figure 5.7).

Interestingly, the expression kinetics of p27 and p21 during cell cycle progression is quite complementary. Whereas in G0 the amount of p27 is high, it subsequently decreases at early-mid G1 due to its degradation. However, the p21 levels that are very low in quiescent cells started to increase just when p27 was decreasing at early-mid G1. These different kinetics are due to the fact that p27 regulates the expression of p21 through the TF Pitx2. It has been shown that p27 represses Pitx2 expression, thus, when p27 is degraded, Pitx2 expression increases and since it is a transcriptional activator of p21, it started to rise at that time [32]. This explains why p21 participates in the recruitment of cyclin D1-Cdk2 complexes at mid-late G1 and not before.

It has to be taken into account that in cells lacking p27, at early G1, cyclin-Cdk complexes could be active, although not recruited on the promoters, and able (perhaps with low efficiency) to phosphorylate p130. In such scenario, transcription of target genes would be facilitated and as a consequence also cell cycle progression. This p130 phosphorylation can occur unless p21 was upregulated due to the absence of p27. In this case p21 could inhibit cyclin-Cdk complexes and block transcription or p130/E2F4 repressed genes or at least to delay it. This phenomenon, can explain why in p27KO cells only the up-regulation of Ets-dependent genes was observed in the expression microarrays. Up-regulation of p21 would block transcription of p130/E2F4 depending genes. Thus, the simultaneous deletion of p27 and p21 strongly induce transcription of targets genes. In this case cyclin-Cdk complexes would be not inhibited and would be more efficient to phosphorylate p130 [93].

### Collaboration between p27 and PCAF in transcriptional regulation

It has also been extensively studied the collaboration between p27 and the co-activator PCAF. The acetyltransferase PCAF [103] has been shown to acetylate a number of proteins involved in cell cycle regulation. Specifically, It acetylates specific lysine residues in the catalytic center of Cdk9 and Cdk2 inducing their inactivation [45, 46]. Since Cdk9 associates with T cyclins and participates in the regulation of transcription by phosphorylating RNA polymerase II, its inactivation induces a transcriptional block. Similarly, PCAF also acetylates lysine residues in Cdk2 leading to its inactivation and thus slowing cell cycle progression. Interestingly, PCAF also acetylates cyclin A at specific lysine residues that stimulates its ubiquitylation and its subsequent degradation [104, 105]. PCAF can also facilitate the nuclear translocation of the Cdk4/6 inhibitor p16^Ink4a^ and inducing Cdk4 inhibition [97]. Thus, PCAF that is considered a transcriptional co-activator also participates in regulation of cell cycle progression by at least three different mechanisms, by degrading cyclin A and by inactivating Cdk4 and Cdk2 all of them involved in the onset and progression of DNA synthesis [106].

Recently, it has been found that PCAF is also able to acetylate p27 at lysine 100. This acetylation induces its ubiquitylation and its degradation via proteasome. During cell cycle PCAF acetylates p27 mostly at early G1 and this acetylation participates in p27 degradation observed at that time [20]. This degradation of p27 facilitates the removing of cyclin D2/D3-Cdk4 from the promoters of target genes as mentioned above (Figure 5). Thus, altogether these data indicates that PCAF plays a role as facilitating cell cycle progression by degrading p27 and blocking Cdk4 activity (when it is not needed) at mid G1. Later on during cell cycle, the induction of Cyclin A degradation and blocking Cdk2 by PCAF facilitates mitosis entry [107]. The transcriptional programs regulated by PCAF and p27 in the colon cancer cell line HCT116 have been recently analyzed by ChIP-seq [76]. 269 protein-encoding genes were found to contain both p27 and PCAF-BSs in their vicinity. The majority of these sites were different for PCAF and p27 indicating the existence of specific distinct chromatin regions able to associate with PCAF or p27. PCAFKO or p27KO cells showed that both regulate the expression of these genes, PCAF as an activator and p27 as a repressor. The double knock down of PCAF and p27 strongly reduced the expression of these genes, indicating that the activating role of PCAF overrides the repressive effect of p27. Interestingly, the transcription factor Pax5 interacts with both p27 and PCAF and its knock down induces the expression of p27/PCAF-TGs indicating that it also participates in the transcriptional regulation mediated by p27/PCAF [76].

### Transcriptional programs regulated by p27

A first approach to the identification of the transcriptional programs regulated by p27 was the analysis of the genes, whose promoters included specific p27-BSs, identified by ChIP on chip and that were functionally classified by GO analysis [73]. Results indicated that the more significant biological processes were: 1) mRNA processing and splicing, 2) translation, 3) cell division, 4) mitochondrial organization and respiration and 5) Golgi vesicle transport. These results suggested that p27 could be directly involved in the regulation of important cellular functions.

A recent expression microarray analysis performed in p27KO-MEFs versus controls deeply amplified the functional aspects that could be regulated by p27 [32]. Specifically, the expression of 4022 genes were altered in p27KO-MEFs, 1844 up-regulated and 2178 down-regulated. GO and KEGG pathway analysis of the altered genes indicated that four of the five biological processes observed to be regulated by p27 in the ChIP on chip experiments: mRNA processing and splicing, cell division, mitochondrial organization and respiration and Golgi vesicle transport were confirmed to be regulated by p27. Moreover, the expression data also confirmed results obtained by ChIP-seq revealing that the main biological processes putatively regulated by p27 as observed by ChIP-seq studies: cell adhesion, neuron differentiation, cell-cell signaling and ion transport were also represented in the expression microarrays. Interestingly, all these studies coincide in that the most significant biological process regulated by p27 is cell adhesion. A KEGG pathway analysis of data from the expression microarray showed different categories involved in cell adhesion as: extracellular matrix-receptor interaction, adherens junction, focal adhesion, gap junction and tight junction with a total of 103 genes, involved in cell adhesion, deregulated in p27KO cells [32]. This analysis confirms that cell adhesion is a major biological process regulated by p27 and this merits to be analyzed in detail in the next future. A recent report showing that cells lacking p27 have a much higher adherence to the culture plates than control cells, strongly support this concept [77].

In addition to that, the KEGG pathway analysis of the expression data revealed a number of very interesting pathways putatively regulated by p27. For instance, in addition to those mentioned above, metabolic pathways as glutathione metabolism, purine and pyrimidine metabolism, fatty acid metabolism, pyruvate metabolism and amino acid metabolism are also regulated by p27. Other functions as axon guidance, mismatch repair and nucleotide excision repair are as well under the control of p27. Another very interesting aspect is that cells lacking p27 deregulates a significant number of genes involved several pathologies as cancer and different neurodegenerative diseases as Huntington's disease, Alzheimer's disease and Parkinson's disease [32].

Finally, it is important the fact that in the absence of p27 a significant number of TFs are deregulated. Specifically, the up-regulation (> 3 fold change) of 42 TFs and the down-regulation (> 3 fold change) of 24 TFs was observed in p27KO cells. The alteration of TF expression in p27KO cells could be responsible for most of the expression changes observed in these cells [32]. The analysis of this list revealed that TFs regulated by p27 are involved in important biological functions. As examples, seven Hoxa genes (Hoxa1-Hoxa7) belonging to a cluster involved in embryo development [108] are up-regulated in p27KO MEFs suggesting a role of p27 in this process [32]. p27 can also participate in patterning and morphogenesis because three of the five Zic homologs (Zic1, 2 and 5) [109] are upregulated in cells lacking p27 [32].

Alteration of TF expression in p27KO brings up the possibility that the around of 4000 genes whose expression is altered in these cells could be a consequence of different transcription waves. First, a limited number of genes directly regulated by p27 would be initially expressed in p27KO cells, and among them, some TFs, as for instance Mef2c, Sox6, Zfpm2 and Shox2 that have p27-BSs in their vicinity [77]. As a consequence, a second transcriptional wave can de generated by the action of these TFs. A number of successive transcriptional waves can be produced thus significantly amplifying the number of genes differentially expressed in p27KO cells.

Remarkably, p27 may be also involved in splicing. A number of specific splicing factors are deregulated in p27KO cells (unpublished results). Interestingly, these results together with those mentioned above regarding the putative regulation of the five major spliceosomal RNAs by p27 suggest an unexpected new role of p27 in this relevant cellular process. However, this putative new role of p27 still remains unexplored.

### Role of p27-mediated transcriptional regulation in Cancer

It is very well described that in many different types of human cancer the decrease of p27 or its cytoplasmic localization is associated with increased malignancy and with a worse outcome [110–114]. Initially, it was assumed that this correlation could be due to the fact that reduced levels of p27 would be unable to efficiently inhibit cyclin-Cdk activities thus the high activity of these kinases would stimulate cell proliferation. However, the new role of p27 as a transcriptional regulator suggested the possibility that in addition to the effect of p27 decrease on cell cycle progression by non-inhibiting cyclin-Cdk complexes, the reduction of p27 would also induce the deregulation of genes that could play a role in tumorigenesis and increased malignancy. In fact, in several cases, it has been stablished a correlation between p27 levels, expression of p27-TGs and decreased survival [73]. On the other hand, the cytoplasmic location of p27 would alter the expression of genes under p27 regulation since transcription is a nuclear function. Additionally, in the cytoplasm p27 plays an oncogenic role by activating cell motility and invasion through modifying the cytoskeleton functions. Specifically, in the cytosol, p27 can associate with RhoA, preventing its activation and thereby modulating actin cytoskeleton dynamics and migration [115, 116]. Thus, p27KO-MEFs have higher RhoA activity, increased numbers of stress fibers and focal adhesions and exhibit a defect in migration [116]. p27 regulates cell migration also by controlling microtubule stability through stathmin or directly by binding to microtubules and promoting microtubule polymerization [117]. It also represses lipid raft trafficking in a stathmin - dependent way. [118]. More recently, cytoplasmic p27 was reported to promote epithelial to mesenchymal transition by binding to JAK2, promoting STAT3 activation and the upregulation of Twist1 [119]. This cytoplasmic behavior of p27 related with cell motility has to be coordinated with the expression of genes involved in cell adhesion that as mentioned above are regulated by p27. Thus, during cell cycle progression when p27 moves from nucleus to the cytoplasm the simultaneous actions of p27 in the cytoplasm and the modifications in the expression of genes under the control of p27 may account for the changes in cytoskeleton activities, cell motility and adhesion. This coordination in normal cells is probably altered in cancer cells with low levels of p27 or with anomalous cytoplasmic localization. So, the role of p27 as a transcriptional regulator might play a relevant role in defining malignancy in tumors. Analysis of the expression microarrays data reveals that the decreased levels of p27 might deregulate a number of genes encoding proteins that participate in the progressive acquisition of biological capabilities by tumors and that define the different hallmarks of cancer [32, 73]. The hallmarks of cancer include 8 biological capabilities acquired during the multiple step development of human tumors. They include sustained proliferation, avoiding growth suppressors, avoiding immune destruction, deregulating cellular energetics, resisting cell death, inducing angiogenesis, enabling replicative immortality and activating invasion and metastasis. Underlying these hallmarks are also genome instability that generates the genetic diversity that expedites their acquisition and inflammation, which fosters multiple hallmark functions [120, 121].

**Figure 6:**
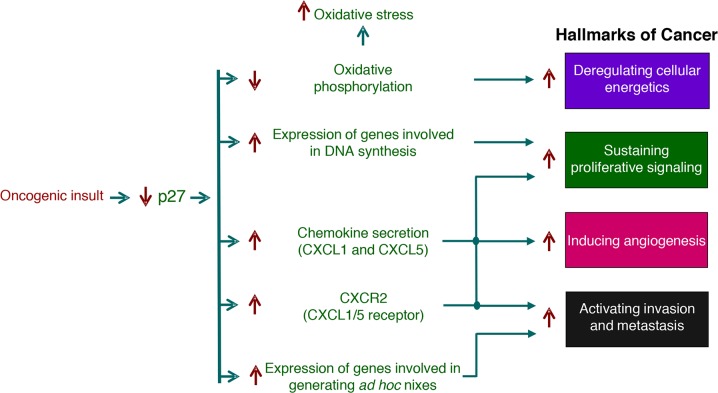
Role of the transcriptional regulatory function of p27 in cancer This scheme summarizes how the decrease of p27 levels observed in many different types of human tumors participates in tumor progression and malignancy. The decrease of p27 produced by oncogenic stimuli induce the deregulation of the expression of genes involved in cellular functions that facilitates the acquisition of tumor capabilities that are defined as Hallmarks of cancer. Specifically, by deregulating genes involved in oxidative phosphorylation and cell proliferation, genes encoding chemokines and their receptors and genes involved in the generation of *ad hoc* metastatic niches, p27 decrease in facilitates tumor progression and malignancy.

The expression microarray data reveal that decreased levels of p27 might deregulate the expression of genes involved in at least four of these cancer hallmarks (Figure 6). One of these Hallmarks is sustained proliferation. Due to its role as a Cdk inhibitor, the decrease of p27 levels will induce an increase in the activity of cyclin-Cdk complexes. Nevertheless, in addition to that it has to be considered that a number of different genes involved in cell proliferation are found deregulated in p27-deficient cells. These deregulated genes include: growth factors, growth factor receptors, proteins that participate in different signaling pathways as G-proteins, adenylate cyclases, phosphodiesterases, different MAPKs and more specifically as mentioned above, the genes involved in DNA replication that are under the control of p130/E2F4 repressor complexes. It merits to specifically mention that different members of the Notch and Wnt pathways are also upregulated [32]. Thus, the decrease of p27 will foster proliferation by deregulating the expression of different signaling pathways.

The decrease of p27 levels can also facilitate the acquisition of a second hallmark: deregulating cellular energetics. Specifically, it has been shown that at least 18 genes involved in oxidative phosphorylation and belonging to different complexes of the respiratory chain are down-regulated in p27KO cells. Interestingly, seven of these genes belongs to complex I. Thus, these results suggest that in p27-deficient cells there is a decrease of oxidative phosphorylation. The decrease of respiration is a key mechanism leading to aerobic glycolysis, a characteristics of tumor cells [122–124]. In addition to that, alterations in a diversity of metabolic enzymes have also been observed [32]. Moreover, it is known that the reduction of respiration of cells in a normoxic environment induce oxidative stress. In these p27 KO cells oxidative stress cannot be efficiently controlled because there is also a significant down-regulation of at least 15 enzymes involved in glutathione metabolism. These data suggest that the detoxifying mechanism involving glutathione should be scarcely effective in these cells [32].

A number of cytokines and chemokines are upregulated in p27KO cells. Interestingly, some of these chemokines as CXCL1 and CXCL5 act through the binding to the CXCR2 receptor that has been involved in angiogenesis, invasion and metastasis [125–127]. Thus, p27 reduction would participate in invasion and metastasis through deregulation of these chemokines but also angiogenesis would be stimulated.

Tumors are thought to arise from mutant stem cells in their native niches. These niches are rich in developmental and self-renewal signals, among them Wnt and TGF-β [128, 129]. Also, their derived progenitors that retain the tumor-initiating capacity and that benefit from these niche signals can also generate tumors [130, 131]. After dissemination, cancer stem cells interact with specialized niches that supports their survival and tumor-initiating potential [132]. These disseminated tumor cells (DTCs) locate themselves in different supportive niches, similar to those that support normal adult stem cells [133]. Additionally, DTCs can set up an *ad hoc* niche by producing specific components as for instance tenascin C, that amplifies Notch and Wnt signaling, TGF-β, which stimulates fibroblasts to secrete periostin, and Procollagen-Lysine, 2-Oxoglutarate 5-Dioxygenase 2 (Plod2) [134–136]. All these components facilitate survival of DTCs thus favoring metastasis. Interestingly, all these proteins of the *ad hoc* niche are under transcriptional repression by p27 [32]. Thus, low levels of p27 would increase the expression of these genes and in such a way facilitate DTC survival and as a consequence metastasis. Finally, a recent report indicates that the TF Sox2 which is transcriptionally repressed by p27 and p21 [91, 137] plays a key role in triggering tumor initiation and cancer-stem cells functions in squamous-cell carcinoma. The targets of Sox2 participate in relevant cellular functions as for instance, stemness, proliferation, survival, adhesion and invasion and metabolism [138]. Considering the broad diversity of cancers expressing Sox2, the functions and downstream targets of Sox2 are likely to be relevant for other cancers. Thus, the progressive decrease of p27 levels in tumors will participate in an increased malignancy by deregulating a number of genes involved in the acquisition of at least 4 cancer hallmarks.

### Role of p27-mediated transcriptional regulation in neurodegeneration

**Figure 7:**
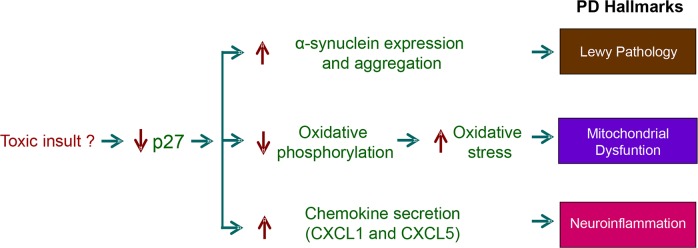
Role of the transcriptional regulatory function of Parkinson's disease (PD) This scheme summarizes how the decrease of p27 levels induce molecular changes similar to those observed in neurons of patients affected by PD. The decrease of p27 induced by not well known mechanisms (environmental toxic agents?), can deregulate the expression of genes that participate in the induction of the main PD Hallmarks. Specifically, of alpha-synuclein (Lewy's pathology), mitochondrial subunits (mitochondrial dysfunction) and chemokines (neuroinflammation).

As mentioned above, one of the relevant transcriptional programs regulated by p27, identified in the expression microarray analysis, is neuron differentiation [32]. This is in agreement with results from the two ChIP-seq analysis reported until now that also identifies neuron differentiation as a major biological process regulated by p27 [76, 114]. Specifically, in the microarray analysis 64 genes of this program were deregulated in p27KO MEFs, 26 of them up-regulated and 38 down-regulated. Interestingly, also 29 genes involved in axon guidance were deregulated in p27KO cells. These genes include 6 semaphorines, 3 ephrins and 4 ephrin receptors. These data clearly indicate that p27 has an active role in the nervous system. However, the more intriguing results were that unexpectedly, several of the transcriptional programs regulated by p27 are related to neurodegenerative diseases as: Alzheimer (AD), Huntington (HD) and Parkinson (PD) [32]. Specifically, in the case of PD the microarrays revealed that the expression of 22 genes related to this disease are altered in cells lacking p27. Interestingly, only two of them are up-regulated whereas the other 20 are down-regulated. One of the upregulated genes is SNCA encoding α-SYN, a major component of the Lewy's bodies (LB) (protein aggregates in specific neurons of affected patients). The presence of LBs is the most relevant neuropathological hallmark of PD [139, 140]. These results clearly suggest that p27 might be involved in the transcriptional regulation of α-SYN. In fact, we have recently reported that actually p27 represses the expression of α-SYN by a mechanism that involves p130/E2F4 complexes [92]. These results clearly indicate that p27 could have a role in the etiology of PD. Moreover, most of down-regulated genes in p27KO cells that are related to PD encode for proteins belonging to many mitochondrial complexes involved in oxidative phosphorylation. Thus, the down-regulation of these genes in p27KO cells suggests a decrease of oxidative phosphorylation and as a consequence and increase in oxidative stress. This can be related to mitochondrial dysfunction that is another relevant hallmark of PD [140]. Neurons from PD patients present a decrease in the activity of the complex I of the respiratory chain and this generates oxidative stress. Finally, the increased expression of chemokines as CXCL1 and CXCL5 in p27KO cells might participate in inducing inflammation that is another PD hallmark [140]. Thus, the decrease of p27 lead to the induction of molecular changes that are similar to those observed in PD (Figure 7).

As oxidative stress and mitochondrial dysfunction are also involved in the induction of AD and HD [141–143], the decreased expression of a significant number of genes involved in oxidative phosphorylation and the increase of chemokine secretion in cells lacking p27 also suggest a role of p27 in the induction of these diseases. Interestingly, a significant downregulation in the expression of ApoE, (~ 15 fold) is also observed in p27KO cells. ApoE is a protein involved in the transport of cholesterol and in the clearance of beta-amyloid (Aβ) peptides from the brain. Aβ peptides derive from proteolysis of the amyloid precursor protein (APP). The fibrillary form of Aβ is the primary component of amyloid plaques found in the brains of AD patients and that are a crucial hallmark of AD [144, 145]. As ApoE is involved in the main mechanism of eliminating Aβ, the strong diminution of ApoE expression in p27KO cells will facilitate Aβ accumulation.

Altogether these data strongly suggest a participation of p27 in the etiology of these neurodegenerative diseases.

## CONCLUSIONS AND PERSPECTIVES

Data accumulated during the last years clearly indicate that in addition to its classical role as a regulator of cyclin-Cdk activity, p27 also plays a role as a transcriptional regulator. This role is mediated by its interaction with specific TFs that facilitates its association with defined chromatin regions. p27 mainly associate with intergenic distal and intronic regions, although it also binds to a significant number of gene promoters. Up to now, a number of TFs that binds to p27 have been reported. It interacts with p130/E2F4 complexes, Ets1, Pax5, Ngn2, AHR, Pax4 and MyoD. It also interacts with the acetyltransferase and transcriptional co-activator PCAF. A mechanistic model of the participation of p27 in the regulation of p130/E2F4 repressed genes has been developed showing that p21 also collaborates in this mechanism. In this model the sequential recruitment of different cyclin-Cdk complexes on the promoters of target genes is accomplished first by p27 and later on by p21. In this model p27 and p21 associate with E2F4/p130 by its carboxyl moiety while they recruit the cyclin-Cdk complexes by the KID domain located at their NH2 half. It can be hypothesized that this model could be extended to other TFs by postulating that p27 can associate with the TF partners by its carboxyl domain and thus in such a way binds to the chromatin. By interacting with cyclin-Cdks by its amino region it facilitates the proximity of the Cdk with substrates associated to the chromatin. However, the extension of this model to other TFs, still remains to be validated. ChIP on chip, ChIP-seq and expression microarray analysis have allowed the identification of the transcriptional programs regulated by p27. In addition to cell cycle progression and functions as mRNA processing and splicing, translation and oxidative phosphorylation, firstly identified by Chip on chip data, a number of other cellular processes as: cell adhesion, cell-signaling, neuron differentiation and ion transport have been identified by ChIP-seq and expression microarrays. Among genes deregulated in p27KO cells there is a number of TFs that probably generates subsequent waves of transcription that, in this case, would not be directly regulated by p27. Interestingly, a reduced number of TFs as Mef2c, Sox6, Zfpm2 and Shox2 that have p27-BSs at their vicinity (as detected by ChIP), are also deregulated in expression microarrays suggesting that they are direct targets of p27. They can be subsequently involved in the triggering of a second transcriptional wave. Many other TFs that do not have p27-BSs in the proximity and that were found deregulated in p27KO cells might be involved in subsequent transcriptional waves.

Finally, altered levels of p27 can participle in different pathologies as cancer and neurodegenerative diseases as PD, AD and HD. In the case of cancer, it is well known that reduced levels of p27 in human tumors are associated with a poor outcome of the patients. The increased malignancy in these tumors can be induced by transcriptional alteration of p27-TGs involved in different cancer hallmarks. Potentially interesting are data indicating that p27 might regulate the expression of genes involved in different neurodegenerative diseases as for instance SNCA, ApoE and genes encoding different subunits of the different oxidative-phosphorylation complexes that could induce oxidative stress. The putative participation of p27, through its transcriptional regulatory role, in the onset and progression of these different neurodegenerative diseases needs to be extensively studied in the next future.
